# Leveraging Artificial Intelligence to Enhance Oncology Care Delivery in Sub-Saharan Africa

**DOI:** 10.1016/j.mcpdig.2026.100337

**Published:** 2026-01-20

**Authors:** Jasmine G. Hughes, Ketan Tamirisa, Ammar Dharani, Faraan Rahim, Erick Mahatara, Ntuli A. Kapologwe, Almotasm Ben Hmeda

**Affiliations:** a Cambridge Brain Tumour Imaging Laboratory, Department of Clinical Neurosciences, University of Cambridge, United Kingdom; b Washington University in St. Louis, MO; c Harvard Medical School, Boston, MA; d School of Medicine, KCMC University, Moshi, Tanzania; e East Central and Southern Africa Health Community (ECSA – HC), Arusha, Tanzania; f London School of Hygiene and Tropical Medicine, University of London, United Kingdom

In sub-Saharan Africa (SSA), with a population of over 1 billion, cancer is one of the leading causes of premature deaths.^1^ There were approximately 820,000 new cases of cancer and 550,000 cancer-related deaths that occurred in SSA in 2022.^2^ The burden of cancer in SSA is expected to double, with an estimate of 1.5 million new cases and 1 million deaths by 2040.^3^ Although there has been an increase in initiatives to address the burden of cancer, there remain several challenges in the process of providing oncology care within SSA. One significant challenge is regarding early diagnosis because delays in receiving a cancer diagnosis can lead to worsening of the condition before receiving treatment and poor treatment outcomes. Costs associated with screening and treatment are another challenge because a significant portion of the SSA population is uninsured and would have to pay out of pocket for medical expenses and consequently pushing them into poverty due to catastrophic health expenditures.^4^ In addition, the limited number of medical personnel, poor planning caused by gaps and weaknesses in the cancer registry system, fragmented regional policies and strategies for cancer management, infrastructure, inadequate primary health care capacity for effective cancer screening, and timely referral and access continue to remain a hurdle for providing oncology care in SSA.^5^^,^^6^ Given these challenges, artificial intelligence (AI)-driven interventions have the potential to be an effective and sustainable solution to improving oncology care in SSA.

## Landscape of AI in Global Oncology

Artificial intelligence can be defined as the development of computer systems equipped to perform tasks comparable with those that require human intelligence. Applications of AI within health care have the potential to not only help physicians provide more efficient care but also lead to an improvement in health outcomes for patients. Artificial intelligence–based diagnostic applications can provide precise diagnoses based on medical imaging and relevant patient information.^7^ Prognostic tools are another type of application, consisting of validated models that are used to predict outcomes for patients.^8^ The utilization of prognostic tools can help guide medical decision making for treatments and resource allocation in limited-resource settings. Other examples of AI applications include remote patient monitoring systems, which can identify potential complications or concerns with patients’ vital signs with real-time feedback to providers.^7^ Current literature suggests a growing trend in the global utilization of AI within oncology, particularly in high income countries.

### Opportunities for AI in Oncology Care in SSA

Recently, Africa has developed key policies and strategies aimed at shaping both the present and future of AI on the continent. In 2024, the African Union endorsed Africa’s Continental AI Strategy, providing a comprehensive roadmap for the responsible and impactful use of AI.^9^ Building on this momentum, the Africa Declaration on Artificial Intelligence was adopted on April 4, 2025, during the inaugural Global AI Summit on Africa held in Kigali, Rwanda.^10^ Today, countries across SSA are at varying stages of implementing AI, reflecting diverse national priorities, capacities, and readiness levels.

Because AI can be used to augment oncology care in each stage of the patient pathway for SSA patients, several of the priorities set for cancer care in SSA, such as increasing cure and improving care, building and maintaining the workforce, innovation and research, and invest in telehealth, can be addressed.^11^ Considering prevention and screening methods, low-cost wearables with the capacity to automate risk prediction based on presentation of symptoms and applications that automate cancer detection in medical imaging can be employed. For a faster and more precise diagnosis and personalized approach to treatment, there is an opportunity to incorporate automated cancer diagnosis and tumor grading through biopsy, pathology, and histology data. This approach could reduce the potential delay in diagnosis and supplement the workload with a limited workforce. Through patient follow-up after treatment management, the potential risk for recurrence can also be predicted and help guide discussions for further interventions.

### Barriers and Challenges

Despite the promise of AI to transform oncology care in SSA, multiple structural and systemic barriers exist that constrain its equitable implementation. For example, across SSA, many hospitals still rely on paper-based records, and the use of EMR systems remains in its preliminary stages.^12^^,^^13^ Despite some efforts by regional organizations such as the East Central and Southern Africa Health Community, cancer registries remain severely underdeveloped. They are often concentrated in urban centers, leaving rural populations invisible in data sets.^14^ This uneven availability creates major blind spots for AI algorithms, which depend on large, representative data sets for training and validation. As a result, models trained on incomplete or biased data risk misclassification and poor generalizability across diverse populations. Cultural and linguistic diversity, spanning over 2000 local languages, adds another barrier because most AI tools are designed for English or French, limiting accessibility and patient trust.^15^

Additionally, infrastructure constraints persist. Although approximately 85% of SSA is covered by mobile broadband signal, only approximately 37% of the SSA population uses the internet, compared with 63% globally.^16^^,^^17^ Even in connected areas, bandwidth is often unstable, and frequent power outages disrupt continuous use of digital systems. High-performance computing infrastructure, such as graphics processing units required for AI training, remains scarce, and hardware maintenance is costly. Without robust digital infrastructure, AI solutions risk remaining pilot projects rather than scalable, sustainable tools.

Sub-Saharan Africa also faces a severe oncology workforce shortage because some countries report having only 1 oncologist per 1 million people, and many African oncologists already manage over 500 new cancer cases per year.^18^ Although AI could potentially help alleviate this shortage, clinician buy-in and effective deployment require substantial training in AI literacy and digital health. Without targeted workforce development and continuous professional support, clinicians may be reluctant to adopt AI tools, or worse, misuse them.

Although SSA accounts for approximately 16% of the global population, the region represents less than 1% of global health expenditure.^19^^,^^20^ Public health systems are already underfunded, and in many parts of SSA, out of pocket payments account for more than 40% of total health expenditure.^21^ This economic reality makes it difficult to sustain AI platforms without external donor funding. Ensuring sustainability will require innovative financing models, public–private partnerships, and integration into national cancer control strategies rather than ad hoc donor-driven initiatives.

### Enablers and Implementation Strategies

#### Digital Infrastructure and Public-Private Partnerships

Several SSA countries are successfully integrating AI into national health strategic plans through public–private partnerships. To ensure efficient, quality and accessible delivery of health services using technology, Rwanda passed a public–private partnership law to foster the development of long-term partnerships with private sector in digital health.^22^ Rwanda’s partnership with Babyl exemplifies the success of this initiative: since 2016, this AI-based triage system has registered over 2 million users and delivered more than 1.3 million virtual consultations, significantly expanding access to health care in rural areas.^22^ In South Africa, Icon Oncology partnered with Limbus AI in 2022 to deploy AI-powered radiation planning software across 27 facilities, reducing treatment preparation time by 80% to 90%.^23^ Similarly, Kenya has implemented AI-powered digital microscopy for cervical cancer screening in resource-limited settings, demonstrating high sensitivity in detecting abnormal cells.^24^

#### Task-Shifting Through AI-Enabled Workforce Development

AI tools are enabling task-shifting strategies to address SSA’s severe oncology workforce shortage. Ethiopia’s HEP Assist, an AI-powered call center developed by Last Mile Health, provides real-time medical guidance to health extension workers, allowing frontline workers to perform screenings, risk assessments, and monitoring that typically require specialists. This system has already reached over 16,000 community health workers, covering 40% of Ethiopia’s network.^25^

#### Local Innovation Ecosystems

Countries with stronger infrastructure such as South Africa, Egypt, Nigeria, and Kenya, are emerging as regional hubs for AI development in oncology. The current AI models focused on malignancies with higher African incidence rates, such as breast, cervical, and colorectal cancers, requires tailoring to local epidemiological and molecular profiles. AI models, which are predominantly trained on data from High Income Countries, can fail to inherently account for SSA’s unique genetic and epidemiological diversity, therefore carrying a high risk of algorithmic bias, which may lead to misdiagnosis, underperformance, and health inequities. Therefore, to ensure AI is a reliable and trusted resource in cancer care, SSA must take action to build and manage their own local, high-quality health datasets and not rely on existing global models. Multisectoral collaborations involving ministries of health, academic institutions, and technology companies are essential for sustainable implementation.

## Conclusion

The implementation of AI-driven interventions has the potential to transform oncology care in SSA. In order to address the barriers to effectively incorporating AI into oncology care in SSA with a sustainable impact, priorities must be set for AI implementation research, which includes evaluating prospective validation models, conducting pilot studies, and cost-effective analysis. There is a pressing need for policies that focus on sustainable funding initiatives for the advancement of AI implementation research. In addition, furthering the development and expansion of innovative ecosystems will be imperative because local hubs can lead the development and expansion of AI-driven applications throughout SSA. The overall process of fostering multisectoral collaborations through global and regional partnerships, having a unified continental policy, ensuring data governance and developing digitally skilled health workforce can lead to a sustainable impact.

## Potential Competing Interests

The authors report no competing interests.

## References

[1] Bray F., Laversanne M., Weiderpass E., Soerjomataram I. (2021). The ever-increasing importance of cancer as a leading cause of premature death worldwide. Cancer.

[2] American Cancer Society (2022). The Burden: Cancer in Sub-Saharan Africa. The Cancer Atlas. Cancer.org.

[3] Bray F., Parkin D.M., African Cancer Registry Network (2022). Cancer in sub-Saharan Africa in 2020: a review of current estimates of the national burden, data gaps, and future needs. Lancet Oncol.

[4] Kaso A.W., Regesu A.H., Haftu H.K. (2025). Magnitude of catastrophic health expenditure and its determinants among cancer patients in low and middle-income countries: a systematic review and meta-analysis. BMC Health Serv Res.

[5] Sharma A., Alatise O.I., O’Connell K. (2021). Healthcare utilisation, cancer screening and potential barriers to accessing cancer care in rural South West Nigeria: a cross-sectional study. BMJ Open.

[6] Oluwasanu M.M., Adejumo P.O., Sun Y. (2024). Challenges and recommendations for improving cancer research and practice in Nigeria: a qualitative study with multi-stakeholders in oncology research and practice. Cancer Control.

[7] Chaturvedi U., Chauhan S.B., Singh I. (2025). The impact of artificial intelligence on remote healthcare: Enhancing patient engagement, connectivity, and overcoming challenges. Intell Pharm.

[8] Tousignant-Laflamme Y., Houle C., Cook C., Naye F., LeBlanc A., Décary S. (2022). Mastering prognostic tools: an opportunity to enhance personalized care and to optimize clinical outcomes in physical therapy. Phys Ther.

[9] (2024). Continental artificial intelligence strategy: harnessing AI for Africa’s development and prosperity. African Union.

[10] Africa declaration on artificial intelligences (2025). Global AI Summit on Africa. https://c4ir.rw/docs/Africa%20Declaration%20on%20Artificial%20Intelligences.pdf.

[11] Ngwa W., Addai B.W., Adewole I. (2022). Cancer in sub-Saharan Africa: a Lancet Oncology Commission. Lancet Oncol.

[12] Kessy E.C., Kibusi S.M., Ntwenya J.E. (2024). Electronic medical record systems data use in decision-making and associated factors among health managers at public primary health facilities, Dodoma region: a cross-sectional analytical study. Front Digit Health.

[13] Serge B., Mbondji E., Humphrey K., Janauschek L. (2024). Health data digitalization in Africa. World Health Organization.

[14] Crocker-Buque T., Pollock A.M. (2015). Appraising the quality of sub-Saharan African cancer registration systems that contributed to GLOBOCAN 2008: a review of the literature and critical appraisal. J R Soc Med.

[15] Alabi J.O., Hedderich M.A., Adelani D.I., Klakow D. Charting the landscape of African NLP: mapping progress and shaping the road ahead. http://arxiv.org/abs/2505.21315.

[16] Björkegren D., Choi J.H., Budihal D.P. Could AI leapfrog the web? Evidence from teachers in Sierra Leone.

[17] Ritchie H., Mathieu E., Roser M., Ortiz-Ospina E. (2023). Internet: our world in data. https://ourworldindata.org/internet.

[18] Mathew A. (2018). Global Survey of Clinical Oncology Workforce. J Glob Oncol.

[19] Sub-Saharan Africa Population (2025). World population review. https://worldpopulationreview.com/continents/sub-saharan-africa.

[20] Kabongo W.N.S., Mbonigaba J. (2024). Public health spending in sub-Saharan Africa: exploring transmission mechanisms using the latent growth curve mediation model. Health Econ Rev.

[21] Voto T.P., Voto B.E., Ngepah N. (2025). The impact of out-of-pocket health expenditure and public health expenditure on poverty in sub-Saharan Africa. Economies.

[22] Nyakabau M.R. (2021). Digital-First Integrated Care: Rwanda’s innovative digital health care service. Transform Health.

[23] Lerm M. (2022). Icon Oncology and Limbus AI™ partner to pioneer radiation AI software in SA. Icon Oncology.

[24] Holmström O., Linder N., Kaingu H. (2021). Point-of-care digital cytology with artificial intelligence for cervical cancer screening in a resource-limited setting. JAMA Netw Open.

[25] Megentta AZ (2025). Equity exemplified through HEP assist: our AI innovation. Last Mile Health.

